# Lomefloxacin Induces Oxidative Stress and Apoptosis in COLO829 Melanoma Cells

**DOI:** 10.3390/ijms18102194

**Published:** 2017-10-20

**Authors:** Artur Beberok, Dorota Wrześniok, Martyna Szlachta, Jakub Rok, Zuzanna Rzepka, Michalina Respondek, Ewa Buszman

**Affiliations:** Department of Pharmaceutical Chemistry, School of Pharmacy with the Division of Laboratory Medicine, Medical University of Silesia, Jagiellońska 4, 41-200 Sosnowiec, Poland; dwrzesniok@sum.edu.pl (D.W.); szlachta.martyna@gmail.com (M.S.); jrok@sum.edu.pl (J.R.); zuzarzepka@gmail.com (Z.R.); michalina.respondek@med.sum.edu.pl (M.R.); ebuszman@sum.edu.pl (E.B.)

**Keywords:** lomefloxacin, melanoma, oxidative stress, DNA fragmentation, apoptosis

## Abstract

Although some fluoroquinolones have been found to exert anti-tumor activity, studies on the effect of these drugs on melanoma cells are relatively rare. The aim of this study was to examine the effect of lomefloxacin on cell viability, reactive oxygen species production, redox balance, cell cycle distribution, DNA fragmentation, and apoptosis in COLO829 melanoma cells. Lomefloxacin decreases the cell viability in a dose- and time-dependent manner. For COLO829 cells treated with the drug for 24, 48, and 72 h, the values of IC50 were found to be 0.51, 0.33, and 0.25 mmol/L, respectively. The analyzed drug also altered the redox signaling pathways, as shown by intracellular reactive oxygen species overproduction and endogeneous glutathione depletion. After lomefloxacin treatment, the cells were arrested in S- and G2/M-phase, suggesting a mechanism related to topoisomerase II inhibition. DNA fragmentation was observed when the cells were exposed to increasing lomefloxacin concentrations and a prolongation of incubation time. Moreover, it was demonstrated that the drug induced mitochondrial membrane breakdown as an early hallmark of apoptosis. The obtained results provide a strong molecular basis for the pharmacologic effect underlying the potential use of lomefloxacin as a valuable agent for the treatment of melanoma in vivo.

## 1. Introduction

Melanoma, the most deadly and aggressive form of skin cancer, derives from pigment-producing cells, namely, melanocytes [1]. These cells are characterized by pro-survival mechanisms that counteract damage-causing factors, including UV radiation [2]. The complex mechanisms of resisting cell death, already active in melanocytes, are further extended in melanoma cells, contributing to the melanoma pro-survival phenotype [1].

Throughout the years, the incidence of melanoma has continued to increase worldwide. According to the predictions of the American Cancer Society, about 90,000 new cases of melanoma will be diagnosed in 2017 [3]. Moreover, the statistics estimate a doubling of melanoma incidence every 10 to 20 years [4]. The prognosis for patients with advanced stages of cutaneous melanoma is poor, with a median survival time of less than one year [5] and a five year survival rate from 5 to 19% [4].

Until 2011, a chemotherapeutic alkylating agent, dacarbazine, was the standard drug for patients with metastatic melanoma, despite its modest efficacy and lack of documented survival benefit [6,7]. Recently, significant progress has been made towards melanoma treatment by approving monoclonal antibodies like ipilimumab, nivolumab, and prembrolizumab, as well as tyrosine kinases inhibitors like vemurafenib, dabrafenib, and trametinib [7,8,9]. However the persisting high toll of deaths resulting from melanoma, as well as a rapid increase in resistance to targeted drugs, is observed [6,7]. Therefore the search for novel chemotherapeutic agents for the treatment of melanoma is still needed.

Lomefloxacin belongs to fluoroquinolones, a class of synthetic antibiotics that are widely used for the treatment of various infections [10]. Their mechanism of bactericidal action involves the inhibition of type II isomerases, namely, DNA gyrase (topoisomerase II) and topoisomerase IV, which areprocaryotic enzymes that are functional analogs of eukaryotic topoisomerase II [11]. By selectively cleaving, rearranging, and religating double helixes, type II topoisomerases are able to relax supercoiled DNA and catalyze the decatenation of interlinked DNA molecules [12]. Fluoroquinolones form stable topoisomerase-drug-DNA complexes and inhibit helix religation. The occurrence of double-stranded DNA breaks disturbs replication and transcription processes, leading to cell death [13,14].

Human topoisomerase II is well validated as a target for some classes of anti-cancer chemotherapeutic drugs, including anthracyclines (doxorubicin, epirubicin, daunorubicin, idarubicin, aclarubicin), mitoxantrone, epipodophyllotoxins (etoposide, teniposide), and amsacrine [15]. These agents act primarily by inhibiting the topoisomerase II-mediated religation of double-strand DNA breaks and thereby inducing apoptosis of cancer cells [15,16]. The clinical use of these antineoplastic drugs is limited by their sensitivity to P-glycoprotein-mediated efflux, as well as to high toxicity [11]. Therefore the search for novel human topoisomerase II inhibitors that avoid resistance due to P-glycoprotein expression is still reasonable.

Cancer cells exhibit higher levels of oxidative stress compared to normal cells. Disproportional increases in intracellular reactive oxygen species (ROS) may selectively induce cancer cell death either through random damaging functions of ROS or by the specific induction of apoptosis via the mitochondrial pathway [17,18]. Several in vitro [19,20] and clinical [21] studies have revealed that fluoroquinolones may cause the depletion of antioxidant enzyme activity and therefore induce significant disruption of the cellular antioxidant status.

Simultaneous topoisomerase II inhibition, apoptosis induction, and oxidative stress generation might be an effective strategy for inducing melanoma cell death. Therefore, we investigated, for the first time, the pro-apoptotic and pro-oxidant activity of lomefloxacin towards the melanotic COLO829 melanoma cell line.

The aim of this study was to examine the effect of lomefloxacin on cell viability, reactive oxygen species production, redox balance, cell cycle distribution, DNA fragmentation, and apoptosis in COLO829 melanoma cells. In order to provide strong experimental evidence for the use of lomefloxacin as a potential drug for the treatment of melanoma, the obtained results were compared with previously received data for normal human melanocytes [22].

## 2. Results

### 2.1. Lomefloxacin Decreases the Viability of COLO829 Cells

**Figure 1:**
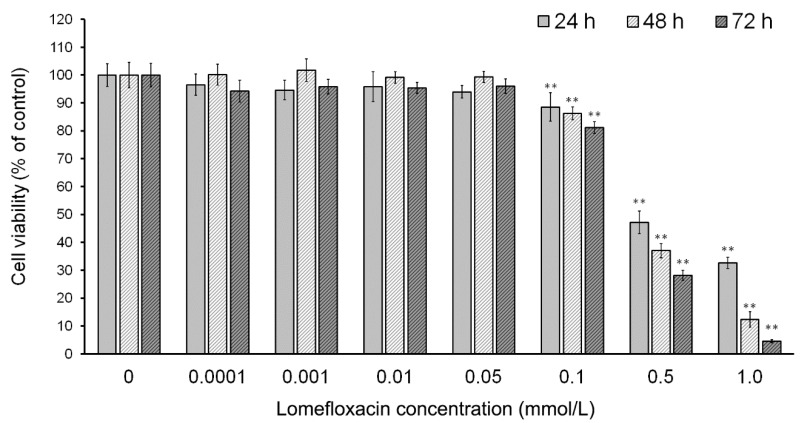
The effect of lomefloxacin on the viability of COLO829 melanoma cells. The cells were treated with various lomefloxacin concentrations (0.0001–1.0 mmol/L) for 24, 48, and 72 h and examined by the WST-1 (4-[3-(4-iodophenyl)-2-(4-nitrophenyl)-2H-5-tetrazolio]-1,3-benzene disulphonate) assay. Data are expressed as percentages of the controls. Mean values ± standard error of the mean (SEM) from three independent experiments (*n* = 3) performed in triplicate are presented. ** *p* < 0.005 versus control samples.

To evaluate the effect of lomefloxacin on the viability of human COLO829 melanoma cells, a WST-1 (4-[3-(4-iodophenyl)-2-(4-nitrophenyl)-2H-5-tetrazolio]-1,3-benzene disulphonate) assay was performed. As shown in Figure 1, the treatment of cells with lomefloxacin concentrations from 0.1 to 1.0 mmol/L for 24 h resulted in the decrease of cell viability by 12 to 68%. The cytotoxic response was more marked after a prolongation of incubation time up to 48 h or 72 h, wherein the viability of COLO829 cells treated with 0.1, 0.5, and 1.0 mmol/L lomefloxacin solutions decreased to 86%, 37%, and 12% or 81%, 28%, and 5%, respectively. After the incubation of the cells with lower drug concentrations (from 0.0001 to 0.05 mmol/L), the loss in cell viability was not statistically significant. For COLO829 cells treated with lomefloxacin for 24, 48, and 72 h, the values of IC_50_ (the concentration of a drug that produces a loss in cell viability of 50%) were found to be 0.51, 0.33, and 0.25 mmol/L, respectively.

### 2.2. Lomefloxacin Induces Morphological Changes in COLO829 Cells

**Figure 2:**
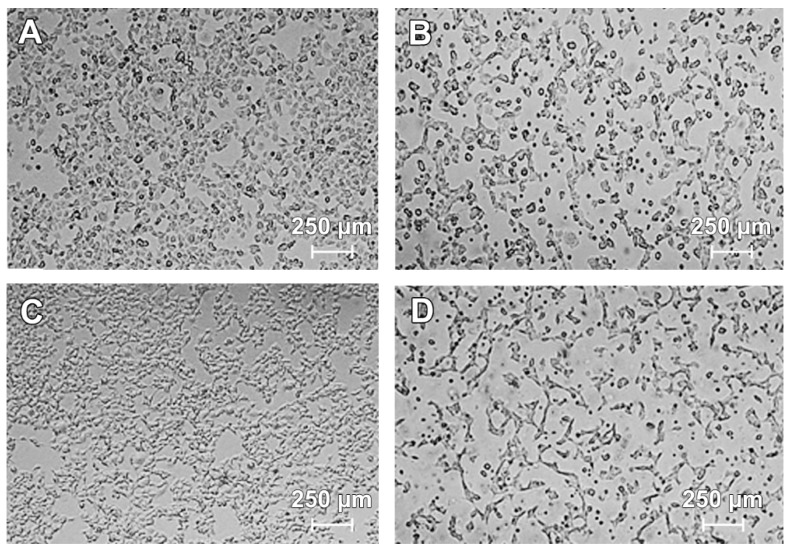

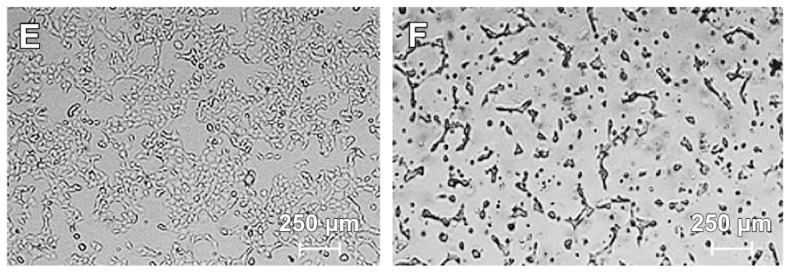
Lomefloxacin induces morphological changes in COLO829 melanoma cells: control COLO829 cells incubated for (**A**) 24 h, (**C**) 48 h, and (**E**) 72 h; cells exposed to lomefloxacin at a concentration of 1.0 mmol/L for (**B**) 24 h, (**D**) 48 h, (**F**) and 72 h. The cells were observed under a light inverted microscope at 40× magnification (scale bar 250 µm).

The morphology of COLO829 cells was estimated by the use of a light inverted microscope at 40× magnification. Figure 2 shows the morphological changes observed in COLO829 cells after incubation with lomefloxacin at a concentration of 1.0 mmol/L for 24, 48, and 72 h. While the untreated cells (Figure 2A,C,E) grew adherently in culture flasks and had regular shapes and sizes, the cells treated with lomefloxacin at a concentration of 1.0 mmol/L for 24, 48, and 72 h (Figure 2B,D,F) became rounded and lost their regular shape and size. Moreover, a loss of cell to cell contact and a decrease in cell number was observed. After 48 and 72 h of incubation with lomefloxacin (Figure 2D,F), most of the COLO829 melanoma cells were detached from their substratum, displaying the typical morphological changes observed during the cell death process.

### 2.3. Lomefloxacin Induces ROS Generation in COLO829 Cells

**Figure 3:**
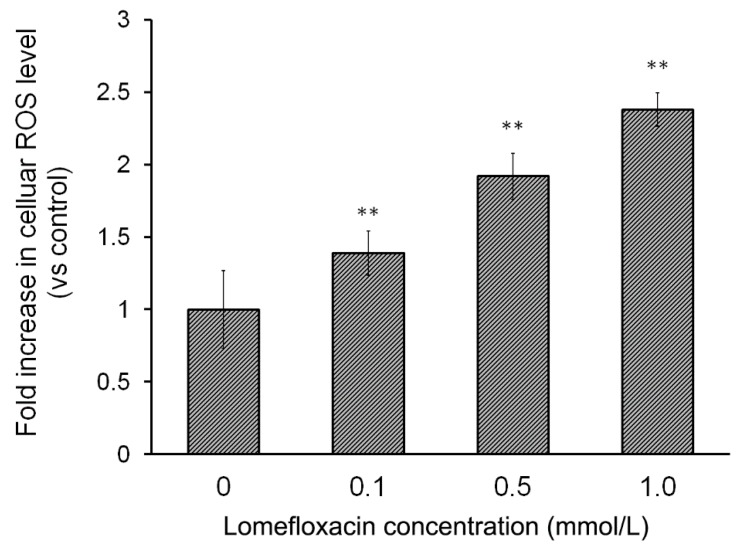
Lomefloxacin induces reactive oxygen species (ROS) production in COLO829 melanoma cells. The cells were exposed to the drug in concentrations of 0.1, 0.5, and 1.0 mmol/L for 24 h. The data are expressed as percentages of the controls normalized to a number of living cells. Mean values ± SEM from three independent experiments (*n* = 3) performed in triplicate are presented. ** *p* < 0.005 versus control samples.

H_2_DCFDA staining was used to detect ROS generation in COLO829 cells exposed to lomefloxacin treatment. As shown in Figure 3, the exposure of COLO829 cells to lomefloxacin leads to ROS overproduction in a concentration-dependent manner. The treatment of cells with lomefloxacin at concenrations 0.1, 0.5, and 1.0 mmol/L for 24 h enhanced ROS production by 38%, 93%, and 137%, respectively, in comparison to the untreated cells (controls).

### 2.4. Lomefloxacin Decreases the Level of Cellular Reduced Glutathione (GSH)

**Figure 4:**
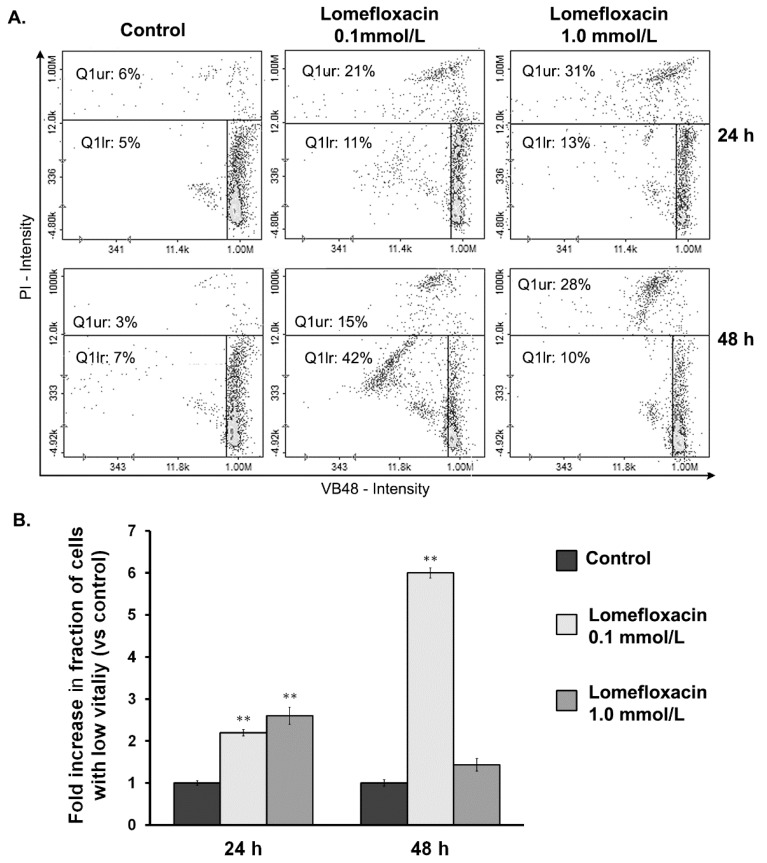
Analysis of the cellular Cellular Reduced Glutathione (GSH) levels in COLO829 cells after exposure to lomefloxacin treatment. (**A**) Histograms presenting the changes of GSH levels in cells exposed to lomefloxacin in concentrations of 0.1 and 1.0 mmol/L. The presented histograms are representative of three independent experiments with similar results. Q1ur are dead cells; Q1lr are cells with low GSH levels (with low vitality). (**B**) The effect of lomefloxacin on cellular GSH levels in COLO829 cells. The cells were treated with lomefloxacin in concentrations of 0.1 and 1.0 mmol/L for 24 and 48 h. Mean values ± SEM from three independent experiments (*n* = 3) performed in triplicate are presented. ** *p* < 0.005 versus control samples.

A decrease in the cellular GSH level is an early sign of the progression of cell death in response to different pro-apoptotic agents. There is a strong correlation between cellular GSH depletion and the progression of apoptosis [23]. This phenomenon seems to be attributed mainly by direct GSH oxidation promoted by ROS. As shown in Figure 4, lomefloxacin caused a cellular decrease in the level of glutathione in its reduced state. Following image cytometric analyses after the exposure of COLO829 cells to lomefloxacin in concentrations of 0.1 and 1.0 mmol/L for 24 h, the percentage of PI (propidium iodide) negative cells with low vitality (with reduced GSH levels) increased from 5 to 11 and 13%, respectively. The response was more marked after the prolongation of the incubation time up to 48 h; for lomefloxacin at a concentration of 0.1 mmol/L, the percentage of cells with reduced GSH levels increased from 7 to 42%. Simultaneously, the treatment of COLO829 cells with lomefloxacin in concentrations of 0.1 and 1.0 mmol/L for 24 and 48 h increased the percentage of PI positive cells (dead cells) from 6 to 31% and from 3 to 28%, respectively.

### 2.5. Lomefloxacin Induces S and G_2_/M-Phase Arrest and DNA Fragmentation in COLO829 Cells

**Figure 5:**
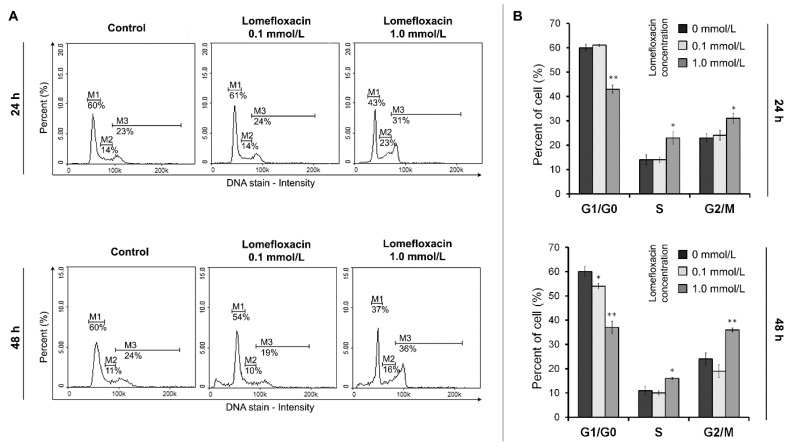
Cell cycle analysis of COLO829 cells exposed to lomefloxacin. (**A**) Histograms presenting the cell cycle distribution in cells exposed to lomefloxacin in concentrations of 0.1 and 1.0 mmol/L for 24 and 48 h. The presented histograms are representative of three independent experiments with similar results. The effect of the drug on the cell cycle distribution was investigated by a fluorescence image cytometer after DAPI (4′,6-diamidino-2-phenylindole) staining. M1G_1_/G_0_ phase; M2—S-phase; M3—G_2_/M phase. (**B**) The effect of lomefloxacin on the cell cycle distribution in COLO829 cells. The cells were treated with lomefloxacin at concentrations of 0.1 and 1.0 mmol/L for 24 and 48 h. Mean values ± SEM from three independent experiments (*n* = 3) performed in triplicate are presented. * *p* < 0.05 versus control samples; ** *p* < 0.005 versus control samples.

**Figure 6:**
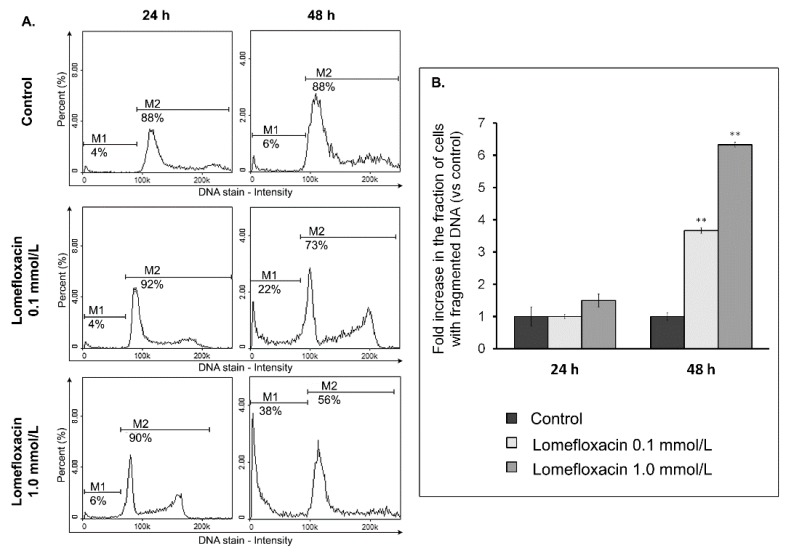
Lomefloxacin induces DNA fragmentation in COLO829 melanoma cells. (**A**) Histograms presenting the induction of DNA fragmentation in cells exposed to lomefloxacin at concentrations of 0.1 and 1.0 mmol/L for 24 and 48 h. The presented histograms are representative of three independent experiments with similar results. The effect of the drug on DNA fragmentation was investigated by a fluorescence image cytometer after DAPI staining. M1—cells having less than one DNA equivalent (so-called sub-G_1_ cells); M2—cells having one or more than one DNA equivalent. (**B**) The effect of lomefloxacin on DNA fragmentation in COLO829 cells. The cells were treated with lomefloxacin at concentrations of 0.1 and 1.0 mmol/L for 24 and 48 h. Mean values ± SEM from three independent experiments (*n* = 3) performed in triplicate are presented. ** *p* < 0.005 versus control samples.

To examine the possible mechanism underlying the cytotoxic activity of lomefloxacin towards COLO829 melanoma cells, the cell cycle changes were estimated using a fluorescence image cytometer. The cells were distributed among three major phases of the cell cycle: G_1_/G_0_ phase (one set of paired chromosomes per cell), S-phase (DNA synthesis with a variable amount of DNA), and G_2_/M phase (two sets of paired chromosomes per cell, prior to cell division). The treatment of COLO829 cells with lomefloxacin at a concentration of 1.0 mmol/L for 24 and 48 h induced significant G_2_/M phase arrest (Figure 5), with the percentages of G_2_/M fraction increasing from 23 to 31% and from 24 to 36%, respectively. Simultaneously, lomefloxacin in the chosen exposure conditions caused a significant increase in the S-phase peak from 14 to 23% and from 11 to 16%, respectively. Moreover, following the image cytometric analyses (Figure 6), lomefloxacin was found to induce DNA fragmentation, the late event in the apoptosis pathway in COLO829 melanoma cells. This phenomenon was demonstrated only for lomefloxacin at concentrations of 0.1 and 1.0 mmol/L and after 48 h of incubation, when the percentages of cells in the sub-G_1_ phase (having less than one DNA equivalent) increased from 6 to 22% and from 6 to 38%, respectively.

### 2.6. Lomefloxacin Disrupts Mitochondrial Transmembrane Potential in COLO829 Cells and Increases the Level of Late-Apoptotic Cells

**Figure 7:**
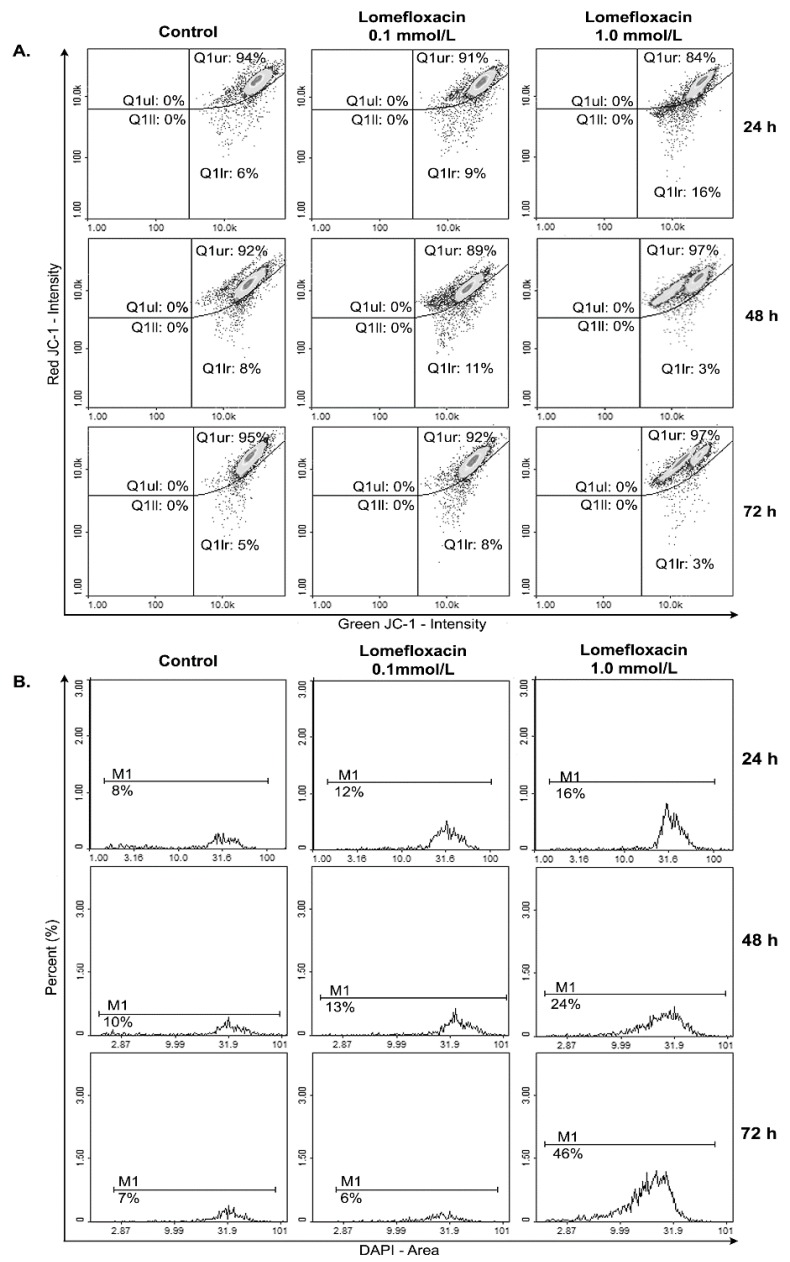
Lomefloxacin induces apoptosis in the COLO829 melanoma cell line. The cells treated with lomefloxacin in concentrations of 0.1 and 1.0 mmol/L for 24, 48, and 72 h were stained with (**A**) JC-1 and (**B**) DAPI dyes and analyzed by a fluorescence image cytometer. The presented histograms are representative of three independent experiments with similar results.

**Figure 8:**
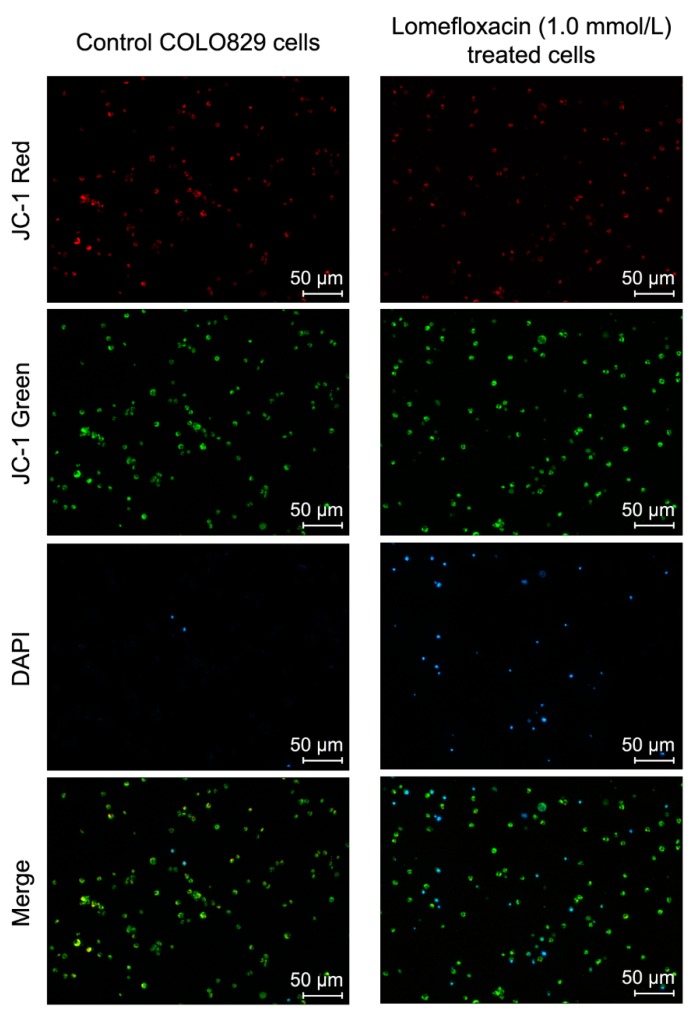
Lomefloxacin induces changes in cellular red and green (JC-1) or blue (DAPI) fluorescence intensity as a result of apoptosis induction in COLO829 cells. The cells were treated with lomefloxacin at a concentration of 1.0 mmol/L for 24 h. The cells were observed under a fluorescence image cytometer (scale bar 50 µm). Q1ur—mitochondrial membrane polarized cells; Q1lr—mitochondrial membrane depolarized/apoptotic cells; M1—DAPI-positive/late apoptotic cells.

A variety of key events during apoptosis involve the mitochondria. Hence, to confirm the involvement of mitochondria in lomefloxacin mediated apoptotic cell death, the mitochondrial membrane potential in lomefloxacin treated COLO829 melanoma cells was estimated. The mitochondrial membrane potential is a crucial parameter of mitochondria function that is used as an indicator of cell death. JC-1 is a lipophilic, cationic dye that can selectively enter mitochondria and reversibly change their color from green to red with increasing membrane potential. In healthy cells with high levels of mitochondria, JC-1 forms complexes with intense red fluorescence, while, in apoptotic cells with low mitochondrial membrane potential, JC-1 remains in the monomeric form, which shows green fluorescence. Following image cytometric analyses (Figure 7 and Figure 8), the percentages of mitochondrial membrane depolarized cells increased only when COLO829 melanoma cells were exposed to lomefloxacin at a concentration of 1.0 mmol/L for 24 h (which increased by 10% compared to the controls). There was no increase in the percentage of depolarized COLO829 cells after the prolongation of incubation time up to 48 h and 72 h. Simultaneously, a significant increase in blue DAPI (4′,6-diamidino-2-phenylindole) fluorescence (Figure 7) was observed, indicating the induction of late-apoptosis after the exposure of COLO829 cells to increasing drug concentrations and the prolongation of the incubation time. The late-apoptotic cell percentages for lomefloxacin treated cells in concentrations of 0.1 and 1.0 mmol/L were 12% and 16% for 24 h of incubation, 13% and 24% for 48 h of incubation, and 6% and 46% for 72 h of incubation, while the values determined for the controls were 8%, 10%, and 7%, respectively.

## 3. Discussion

Despite the recent progress in melanoma therapies, many metastatic melanoma patients still demonstrate a high mortality risk. The aggressive nature of this type of cancer, the occurrence of drug resistance, and severe side effects associated with currently used therapies are the cause of an urgent need for effective anti-melanoma agents [24].

Fluoroquinolone derivatives display activity, not only against bacterial topoisomerases, but also against eukaryotic topoisomerases and thus exert toxicity to mammalian cancer cell lines and in vivo tumor models [25]. The novel quinolone-based topoisomerase II inhibitor, called Vosaroxin (syn. Voreloxin), is under phase III clinical trials to treat the patients with acute myelogenous leukemia and ovarian cancer [11,26]. Nevertheless, several in vitro studies have shown that well known fluoroquinolone derivatives like ciprofloxacin, levofloxacin, enoxacin, ofloxacin, fleroxacin, moxifloxacin, and gatifloxacin induce apoptosis and cell cycle arrest in various cancer cell lines, e.g., bladder carcinoma, transitional carcinoma, and colon carcinoma cells [27,28,29,30]. Fluoroquinolone antibiotics may thus be considered an exploitable source of potent anticancer drugs with already established pharmacokinetic characteristics, which makes them unique among other classes of antibiotic members.

Previously we have demonstrated that lomefloxacin [31], in comparison to other fluoroquinolone derivatives like norfloxacin and moxifloxacin [20], possesses the highest capacity to induce oxidative stress in normal human melanocytes. The depletion of antioxidant status was demonstrated by the ability of the drug to alter the activity of the cellular antioxidant enzymes superoxide dismutase (SOD), catalase (CAT), and glutathione peroxidase (GPx), which indicates that lomefloxacin triggers the generation of ROS, mainly superoxide radical anion and hydrogen peroxide. This phenomenon may be explained by the nature and position of the substituents attached to the quinolone ring [32]. Halogenation (chlorine, fluorine) of position 8, in concert with fluorination of position 6 (the so-called double-halogenated quinolones), has demonstrated a strong ability to produce ROS. Therefore, lomefloxacin and sparfloxacin have been reported to have relatively high toxic potential compared with other fluoroquinolones, e.g., ciprofloxacin or norfloxacin [33].

On the basis of limited in vitro and in vivo data documenting the potential cytotoxic effect of lomefloxacin, and because the molecular mechanism underlying the anticancer action of this drug is not elucidated, we investigated the effect of lomefloxacin on the human melanoma cell line. In this study, we determined for the first time the impact of the drug on cell viability, ROS generation, the level of GSH, cell cycle distribution, and the apoptosis pathway in human melanotic COLO829 melanoma cells.

Lomefloxacin was found to decrease the viability of COLO829 melanoma cells in a time- and concentration-dependent manner (Figure 1). After the incubation of cells with lomefloxacin for 24 h, a decrease in cell viability was observed for higher drug concentrations (from 0.1 to 1.0 mmol/L) up to 32% of the control. The cytotoxic response was more marked when the cells were incubated with lomefloxacin for 48 and 72 h. At these conditions, the drug at the highest analyzed concentration (1.0 mmol/L) decreased the viability of COLO829 cells by 88% and 95%, respectively. The cell morphology assessment indicated that cells treated with lomefloxacin became rounded and lost their cell-cell contact (Figure 2). Moreover, cell shrinkage, reduced cell volume, and irregularities in cell contour and size, which are all well known apoptotic characteristics, were noticed. The observed changes in cell morphology were found to be irreversible, suggesting that the cells were programmed to die when treated with lomefloxacin. Perucca et al. [34] analyzed the cytotoxic effect of lomefloxacin in the epithelial cancer cell lines human cervix carcinoma (HeLaS3) and squamous carcinoma (A431). The authors demonstrated that lomefloxacin in the highest analyzed concentration (0.1 mmol/L) reduced the number of A431 cells by about 30% after 24 h incubation. Simultaneously, this drug had no effect on the viability of HeLaS3 cells, which indicates that lomefloxacin is similar and more cytotoxic to A431 and HeLaS3 epithelial cancer cells. Previously we have demonstrated that the treatment of normal human epidermal melanocytes with lomefloxacin for 24 h decreased the cell viability in a concentration-dependent manner [22]. The exposure of cells to lomefloxacin in concentrations from 0.1 to 1.0 mmol/L decreased the cell viability by 17 to 74%. The main difference between the cytotoxicity of lomefloxacin towards COLO829 melanoma cells and towards normal human melanocytes was observed when the IC_50_ values were compared. The value of IC_50_ determined for normal human melanocytes was found to be 0.75 mmol/L [22], which is higher than the value of this parameter established for COLO829 melanoma cells, 0.51 mmol/L. The obtained results indicate the higher sensitivity of melanoma cells than normal melanocytes to lomefloxacin treatment. Baharara et al. [35] demonstrated that dacarbazine in a concentration of 8.0 mmol/L caused a 50% decrease in the viability of the B16F10 melanoma cell line after 48 h of incubation. In our study, about a 50% decrease in the viability of COLO829 cells was achieved for lomefloxacin at a concentration of 0.5 mmol/L (IC_50_) and 24 h of incubation, which indicates that lomefloxacin is more cytotoxic towards melanoma cells than dacarbazine is.

The overproduction of ROS is a well-known mediator in the signal transduction of apoptosis. Increased levels of ROS can induce oxidative stress, a loss of cell function, and finally lead to cell death, that is, apoptosis. It can also induce lipid peroxidation and the cross-linking of thiol groups in proteins [36]. Moreover, tumors can be sensitized to chemotherapy and other antitumor treatment by disabling antioxidant defenses (nicotinamide adenine dinucleotide phosphate (NADPH) and GSH) through metabolic inhibition. Hence, strategies aimed at altering redox signaling in tumor cells and intended to disable key antioxidant systems in the presence of ROS inducers might represent promising new anticancer treatments [23]. The present study was carried out to examine whether lomefloxacin could induce ROS overproduction in COLO829 melanoma cells. The obtained results demonstrate that lomefloxacin induces oxidative stress in cells. Elevated levels of intracellular ROS were observed, especially when the COLO829 cells were exposed to the analyzed drug in the highest concentration, i.e., 1.0 mmol/L. The percentage of ROS increased to 237% compared to the control (Figure 3), which indicates that the observed cytotoxic effect of lomefloxacin towards COLO829 cells may be associated with increased levels of ROS production.

The intracellular redox status is a precise balance between the oxidative stress and endogenous thiol buffers present in the cells. When unbalanced, it could trigger cellular events downstream such as alterations in mitochondrial function and cell signaling pathways, which lead to apoptotic cell death [17]. Many anticancer drugs can induce apoptosis by disrupting the redox balance. Since GSH, the most abundant cellular thiol and the major determinant of the cellular redox equilibrium, has been shown to be an important factor in apoptosis [37], in the present study, we examined whether the cytotoxic response of lomefloxacin towards COLO829 cells might be associated with the depletion of intracellular GSH levels. The fluorescence image cytometer analysis revealed that lomefloxacin increases the percentages of cells with low GSH levels (Figure 4). The six-fold increase in the percentage of cells with reduced GSH levels was stated for the drug at a concentration of 0.1 mmol/L and 48 h incubation time. At higher drug concentrations (1.0 mmol/L), lomefloxacin caused a significant increase in the percentages of PI-positive (dead) cells, wherein a large number of cells with low vitality lost their plasma membrane integrity. Mirkovic et al. [38] have demonstrated that resistance to radiation-induced apoptosis in a mouse lymphoma cell line was reversed by depleting the cellular thiol levels. Moreover, the depletion of GSH rendered the cells more sensitive to apoptotic agents [39]. On the other hand, elevated GSH levels may decrease apoptosis either by facilitating DNA repair [40] or by buffering the drug-induced oxidative stress [41]. These findings suggest that lomefloxacin may trigger apoptosis in COLO829 cells by depleting the intracellular thiol levels.

The ability of lomefloxacin to induce apoptosis was further confirmed by cell cycle and DNA fragmentation analysis in COLO829 cells. Cell cycle changes have been reported as an important marker that confirms apoptosis [42]. The analyzed drug caused both S- and G_2_/M-phase cycle arrest in COLO829 cells (Figure 5), suggesting a mechanism related to topoisomerase II inhibition. Moreover, as evidenced by the image cytometry analysis of DNA fragmentation, lomefloxacin was found to induce oligonucleosomal DNA fragmentation, confirming the induction of apoptosis in COLO829 melanoma cells (Figure 6). The demonstrated increase in ROS generation in COLO829 cells after lomefloxacin treatment may enhance the ability of the analyzed drug to induce this key event of apoptosis. The four-fold and six-fold increases in the percentages of cells with fragmented DNA was observed when the cells were exposed to lomefloxacin in concentrations of 0.1 mmol/L and 1.0 mmol/L for 48 h. These findings are in agreement with the data demonstrated by Perruca et al. [34], wherein lomefloxacin in lower concentrations induced S- and G_2_/M-phase cycle arrest in A431 or HeLaS3 cells, whereas, in the case of higher drug concentrations, the accumulation of cells in the sub-G_1_-phase was stated. Yadav et al. [29] showed the ability of another fluoroquinolone derivative, ciprofloxacin, to induce S-phase arrest as well as DNA fragmentation in human pancreatic cancer cells. In contrast, Kloskowski et al. [43] demonstrated that, in cases of human non-small lung cancer cells, ciprofloxacin caused cell cycle arrest at the G_2_/M checkpoint. The differences in the mechanistic action of the fluoroquinolone derivative could be attributed to the difference in the origin of the cell type. Moreover, it was stated that different molecular pathways can be activated by various fluoroquinolone derivatives in the same cell line [44].

There is no data focusing on the effects of fluoroquinolones on human melanoma cell lines. It is assumed that fluoroquinolones inhibit bacterial type II topoisomerase (gyrase DNA); however they can also affect the viability of certain eukaryotic cells [19,20,22,23,28,29]. It is hypothesized that these effects may occur possibly via the unselective inhibition of mitochondrial DNA synthesis with subsequent mitochondrial injury [45]. Thus, topoisomerase inhibitors may induce the selective loss of mitochondrial DNA, finally leading to disorders in the respiratory chain and the depletion of intracellular adenosine triphosphate (ATP) stores. Energy depletion favours apoptosis via the induction of cell cycle arrest in the S-phase and/or G_2_/M phase. Therefore, we investigated mitochondria-dependent events during apoptosis in human melanoma cells such as the breakdown of mitochondrial membrane potential.

Apoptosis may be initiated by the stimulation of death receptors located on the cell surface or through an intrinsic pathway involving the release of apoptotic signals from mitochondria [46]. In the present study, we have demonstrated for the first time that lomefloxacin induces apoptosis in COLO829 melanoma cells as a result of mitochondrial membrane breakdown (Figure 7 and Figure 8). An almost three-fold increase in the percentage of depolarized cells (results from JC-1 staining) was observed for the drug in a concentration of 1.0 mmol/L and 24 h of incubation. This phenomenon may be explained by the fact that the disruption of mitochondrial transmembrane potential is an early hallmark in the apoptosis pathway. Moreover, it is possible that lomefloxacin may trigger apoptosis via both intrinsic and extrinsic pathways. After the incubation of the cells with lomefloxacin up to 48 and 72 h, an increase in the percentages of depolarized cells was not observed. Simultaneously, the percentage of late-apoptotic COLO829 cells (results from DAPI-staining) increased, reaching the maximum lomefloxacin concentration (1.0 mmol/L) and 72 h of incubation time (an increase of 40%) (Figure 7 and Figure 8).

**Figure 9:**
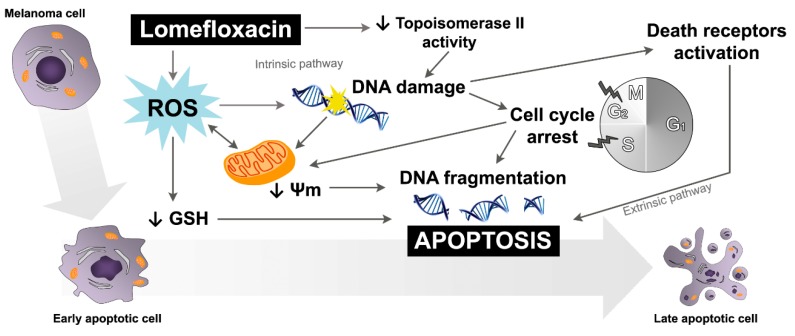
Schematic diagram showing the potential mechanism underlying apoptosis induction in COLO829 melanoma cells by lomefloxacin; Ψm—mitochondrial transmembrane potential.

The obtained data suggest that both the intrinsic pathway, initiated at the mitochondria, and the extrinsic pathway, initiated by binding of specific ligands generated through ROS overproduction and DNA damage, may be involved in lomefloxacin-induced apoptosis in COLO829 melanoma cells (Figure 9). Similar conclusions for human pancreatic cancer cells were drown by Yadav et al. [29], wherein ciprofloxacin induced extrinsic as well as intrinsic mitochondrial apoptotic pathways.

One has to take into consideration that the lomefloxacin concentrations found to have cytotoxic and pro-apoptotic effects on COLO829 cells are about 10-fold higher than the concentrations normally observed in clinical trials after two *po* doses of 800 mg [47]. However, the concentrations of lomefloxacin in the targeted tissues may be significantly higher than the concentrations observed in the serum. In other studies describing the effect of ciprofloxacin on human androgen independent prostate carcinoma PC3 cells [48], the colon carcinoma cell lines CC-531, SW-403, and HT-29 [27], as well as the pancreatic cancer cell lines MIA PaCa-2 and Panc-1 [29], the drug was used even in concentrations of 400 µg/mL (above 1.0 mmol/L). In all the mentioned studies, significant decreases in cell viability and the induction of apoptosis were shown for ciprofloxacin concentrations higher than 200 µg/mL (about 0.5 mmol/L). In our study, significant changes in the analyzed parameters were observed for lomefloxacin concentrations lower than 0.5 mmol/L, especially when COLO829 cells were exposed to the drug for 48 h. Moreover, we have previously demonstrated that lomefloxacin forms complexes with melanin, which may lead to the accumulation of this drug in melanin reach tissues [22]. The obtained results showed that the absolute amount of a drug bound to melanin (expressed as μmol/mg) increases when the initial drug concentration rises and the incubation time is prolonged. The performed Scatchard plot analysis of drug-melanin binding has shown that at least two classes of independent binding sites participate in the lomefloxacin-melanin complex formation: strong binding sites with the association constant K_1_ ~ 10^5^ M^−1^ and weak binding sites with the association constant K_2_ ~ 10^2^ M^−1^. The total number of binding sites (n_1_ + n_2_) was 0.92 μmol lomefloxacin per 1 mg melanin. The affinity and capacity for the binding of lomefloxacin to melanin are similar to those of chloroquine, which is well known for its high affinity for melanin [49,50]. Thus, it is possible that lomefloxacin concentrations in COLO829 cells may be significantly higher than those in serum, and, therefore, the cytotoxic response, as well as the induction of apoptosis in the presence of this drug, could be observed in vivo.

## 4. Materials and Methods

### 4.1. Chemicals

Lomefloxacin hydrochloride was purchased from Sigma-Aldrich Inc. (St. Louis, MI, USA). Growth medium RPMI 1640, amphotericin B, penicillin, streptomycin, fetal bovine serum, and trypsin/EDTA (ethylenediaminetetraacetic acid) were obtained from Cytogen (Srebrna, Poland). Cell Proliferation Reagent WST-1 was purchased from Roche GmbH (Mannheim, Germany). Solutions 3 (1 µg/mL DAPI, 0.1% triton X-100 in phosphate-buffered saline (PBS)), 7 (200 µg/mL JC-1), 8 (1 µg/mL DAPI in PBS), and 5 (VB-48^TM^, propidium iodide—PI, acridine orange—AO), NC-Slide A8, and Via-1-Cassette (AO and DAPI fluorophores) were obtained from ChemoMetec (Allerod, Denmark). The remaining chemicals were produced by POCH S.A. (Gliwice, Poland).

### 4.2. COLO829 Melanoma Cell Culture

The human melanotic melanoma cell line, COLO829, was obtained from the American Type Culture Collection (ATCC) (CRL-1974, Manassas, VA, USA). The cells were cultured in Roswell Park Memorial Institute medium (RPMI) 1640 medium (with l-glutamine), supplemented with 10% fetal bovine serum, penicillin (10,000 U/mL), streptomycin (10 mg/mL), and amphotericin B (0.25 mg/mL), at 37 °C in 5% CO_2_. All the experiments were performed using the cells in passages 8 to 11.

### 4.3. Cell Viability Assay

The viability of COLO829 melanoma cells was evaluated by the WST-1 (4-[3-(4-iodophenyl)-2-(4-nitrophenyl)-2H-5-tetrazolio]-1,3-benzene disulphonate) colorimetric assay. The rate of WST-1 cleavage by mitochondrial dehydrogenases correlates with the number of viable cells. In brief, 2500 cells per well were placed in a 96-well microplate in a supplemented RPMI 1640 growth medium and incubated at 37 °C and 5% CO_2_ for 24 h. After incubation, the medium was removed, and the cells were treated with lomefloxacin solutions in concentrations ranging from 0.0001 mmol/L to 1.0 mmol/L. After 21, 45, or 69 h of incubation, 10 μL of WST-1 solution was added to 100 μL of culture medium in each well, and the incubation was continued for 3 h. The absorbance of the samples was measured at 440 nm using a reference wavelength at 650 nm, against the controls (the same cells but not treated with lomefloxacin), using a microplate reader Infinite 200 Pro (Tecan, Männedorf, Switzerland). The controls were normalized to 100% for each assay, and the treatments were expressed as a percentage of the controls.

### 4.4. Cell Morphology Assessment

In order to investigate the effect of lomefloxacin on cell morphology, the COLO829 cells were seeded in T-25 flasks (1 × 10^6^ cells/flask) in RPMI 1640 supplemented medium. Treatment with lomefloxacin at a concentration of 1.0 mmol/L began 24 h after seeding. After 24, 48, and 72 h, the cells were examined under alight inverted microscope from NIKON TS100F (Tokyo, Japan).

### 4.5. H_2_DCFDA Cellular ROS Detection Assay

The oxidation of 2,7-dichlorodihydrofluorescein diacetate (H_2_DCFDA) into 2,7-dichlorofluorescein (DCF) was used to assess the ROS generation in COLO829 melanoma cells after lomefloxacin treatment. In brief, 2500 cells per well were placed in a 96-well dark microplate in a supplemented RPMI 1640 growth medium and incubated at 37 °C and 5% CO_2_ for 24 h. After incubation, the medium was removed and the cells were treated with lomefloxacin solutions ranging from 0.1 mmol/L to 1.0 mmol/L. After 24 h of treatment, the medium was removed and the cells were incubated with 10 µM H_2_DCFDA for 30 min at 37 °C and washed twice with PBS to remove excess dye. The fluorescence was read at wavelengths of 485 nm of excitation and 530 nm of emission using a microplate reader Infinite 200 Pro (Tecan, Switzerland). The obtained results, normalized to a number of living cells, were finally expressed as a percentage of the controls.

### 4.6. Vitality Assay—Assessment of the Level of Cellular Reduced Glutathione (GSH)

The analysis of the level of cellular thiols in COLO829 cells was performed by the use of the fluorescence image cytometer from NucleoCounter NC-3000 (Allerod, Denmark). The COLO829 cells were seeded in T-75 flasks at a density of 2 × 10^6^ cells per flask. The treatment with lomefloxacin in concentrations 0.1 mmol/L and 1.0 mmol/L began 24 h after seeding. After 24 or 48 h of incubation, the cells were harvested by trypsinization, loaded into Via-1-Casette, and counted using a NucleoCounter NC-3000 image cytometer. 1.0 × 10^6^ cells were suspended in 1.0 mL of culture medium, and one volume (10 µL) of Solution 5 (which contains AO that stains all cells, PI that stains dead cells only, and VB-28^TM^ that stains viable cells in an intensity-dependent manner to the level of thiols) was added into 19 volumes (190 µL) of the cell suspension. The stained cells were loaded into an NC-Slide A8 and measured using the Vitality assay protocol in the NucleoCounter NC-3000 image cytometer. The obtained histograms were used to demarcate the percentages of PI negative cells with low vitality (low level of cellular thiols), PI negative cells with high vitality (healthy cells), and PI positive cells (dead cells).

### 4.7. Fixed Cell Cycle-DAPI/DNA Fragmentation Assay

The cell cycle and DNA fragmentation analysis of the COLO829 cells was performed by the use of the fluorescence image cytometer from NucleoCounter NC-3000 (Denmark). The COLO829 cells were seeded in T-75 flasks at a density of 2 × 10^6^ cells per flask. Lomefloxacin treatment in concentrations of 0.1 mmol/L and 1.0 mmol/L began 24 h after seeding. After 24, 48, or 72 h of incubation, the cells were harvested by trypsinization, loaded into Via-1-Casette, and counted using a NucleoCounter NC-3000 image cytometer. 1.5 × 10^6^ cells were suspended in 0.5 mL PBS and fixed with 4.5 mL of 70% cold ethanol for at least 2 h for the analysis of cell cycle distribution and for at least 12 h for the analysis of DNA fragmentation. Then the ethanol was removed, and the cells were re-suspended in PBS and centrifuged for 5 min at 500× *g*. To each sample, 0.5 mL of Solution 3 (which, in additon to DAPI, contains 0.1% triton X-100 to trigger cellular membrane damage) was added, and the cell pellets were incubated for 5 min at 37 °C. The stained cells were loaded into an NC-Slide A8 and measured using Fixed Cell Cycle-DAPI/ DNA fragmentation assay protocols in the NucleoCounter NC-3000 image cytometer. The obtained DNA content histograms were used to demarcate cells in different cell cycle stages or to demarcate apoptotic cells with fragmented DNA having less than one DNA equivalent (so-called Sub-G_1_ cells).

### 4.8. Mitochondrial Potential Assay

The mitochondrial transmembrane potential (∆Ψm) was measured by the use of the fluorescence image cytometer NucleoCounter NC-3000 (Denmark). The COLO829 cells were seeded in T-75 flasks at a density of 2 × 10^6^ cells per flask. Lomefloxacin treatment in concentrations of 0.1 mmol/L and 1.0 mmol/L began 24 h after seeding. After 24, 48, or 72 h of incubation, the cells were harvested by trypsinization, loaded into Via-1-Casette, and counted using a NucleoCounter NC-3000 image cytometer. To 1.0 × 10^6^ cells, 12.5 µL of Solution 7 was added, and the cells were incubated for 15 min at 37 °C. The stained cells were then centrifuged at 400× *g* for 5 min and washed twice with PBS. The cell pellets were re-suspended in 0.25 mL of Solution 8 (which contains DAPI in PBS) and analyzed immediately using the NC-Slide A8 and Mitochondrial Potential Assay protocol in the NucleoCounter NC-3000 image cytometer. The cellular blue (DAPI), green, and red (JC-1) fluorescence was quantified. The cells with collapsed mitochondrial potential exhibited a decrease in their red/green fluorescence intensity ratio. The obtained scatter-plots and histograms were used to demarcate the percentages of polarized/healthy cells, depolarized/apoptotic cells, and DAPI positive/late apoptotic or necrotic cells.

### 4.9. Statistical Analysis

In all experiments, the mean values of at least three separate experiments (*n* = 3) performed in triplicate ± standard error of the mean (SEM) were calculated. The results were analyzed statistically using GraphPad Prism 6.01 Software by means of a one-way ANOVA, as well as Dunnett’s comparison test. In all cases, the statistical significance was found for *p*-values lower than 0.05.

## 5. Conclusions

The induction of apoptosis is the main mechanism through which a number of chemotherapeutic agents inhibit cancer cell proliferation. The obtained results provide strong experimental evidence indicating the high cytotoxic activity of lomefloxacin towards COLO829 melanoma cells. The cytotoxic response was found to be mediated by the disruption of redox balance, S- and G_2_/M-phase cycle arrest, the breakdown of the mitochondrial membrane potential, and the induction of DNA fragmentation, which trigger drug-induced apoptosis in COLO829 cells (Figure 9). Moreover, when compared with normal human melanocytes, lomefloxacin demonstrates higher cytotoxic activity towards COLO829 melanoma cells than do normal pigmented cells. To the best of our knowledge, this provides, for the first time, an important molecular basis for the pharmacologic effect underlying high lomefloxacin cytotoxic activity towards human melanoma cells and gives a new insight into the therapeutic properties of this drug. The obtained results also support the hypothesis that 6,8-difluoroquinolones may be considered a class of drugs with targeted effects on tumor cells and may constitute a basis for the development of new fluoroquinolone derivatives with potential anticancer activity [34]. With further investigation regarding lomefloxacin preclinical and clinical efficacy, the presented findings could be considered as a potential anti-melanoma therapy.
